# Tuning the charge of polyelectrolyte complex membranes prepared *via* aqueous phase separation†

**DOI:** 10.1039/d1sm01199e

**Published:** 2021-09-30

**Authors:** Elif Nur Durmaz, Joshua D. Willott, Md Mizanul Haque Mizan, Wiebe M. de Vos

**Affiliations:** Membrane Science and Technology, MESA+ Institute for Nanotechnology, University of Twente, Faculty of Science and Technology P.O. Box 217 7500 AE Enschede The Netherlands w.m.devos@utwente.nl

## Abstract

In this work, polyelectrolyte mixing ratio is studied as a tuning parameter to control the charge, and thus the separation properties of polyelectrolyte complex (PEC) membranes prepared *via* Aqueous Phase Separation (APS). In this approach, various ratios of poly(sodium 4-styrenesulfonate) (PSS) and poly(diallyldimethylammonium chloride) (PDADMAC) are mixed at high salinity and the PEC-based membranes are then precipitated using low salinity coagulation baths. The monomeric ratio of PSS to PDADMAC is varied from 1.0 : 0.8 through to 1.0 : 1.2. Obtained membranes have an asymmetric structure and function as nanofiltration membranes with on average 1 L m^−2^ h^−1^ bar^−1^ pure water permeance and <400 Da molecular weight cut-off (MWCO); except for the 1.0 : 1.2 membrane, where the water permeance was much higher (>20 L m^−2^ h^−1^ bar^−1^) with a similarly low MWCO. For the first time, we report the formation of both negatively and positively charged PSS–PDADMAC based APS membranes, as determined by both streaming potential and salt retention measurements. We hypothesize that the salt type used in the APS process plays a key role in the observed change in membrane charge. The point where the membrane charge transitions from negative to positive is found to be between the 1.0 : 0.9 and 1.0 : 1.0 PSS : PDADMAC ratios. The polyelectrolyte ratio not only affects membrane charge, but also their mechanical properties. The 1.0 : 0.9 and 1.0 : 1.0 membranes perform the best amongst the membranes prepared in this study since they have high salt retentions (up to 90% Na_2_SO_4_ and 75% MgCl_2_, respectively) and better mechanical stability. The higher permeance of the more charged, and thus more swollen, 1.0 : 0.8 and 1.0 : 1.2 membranes provide a relevant new direction for the development of APS-based PEC membranes.

## Abstract 2

Positively and negatively charged membranes are obtained by simply changing the mixing ratio of the polyelectrolytes in a fully aqueous preparation approach. This leads to selective retention of asymmetric salts and a new membrane tuning parameter.

## Graphical abstract

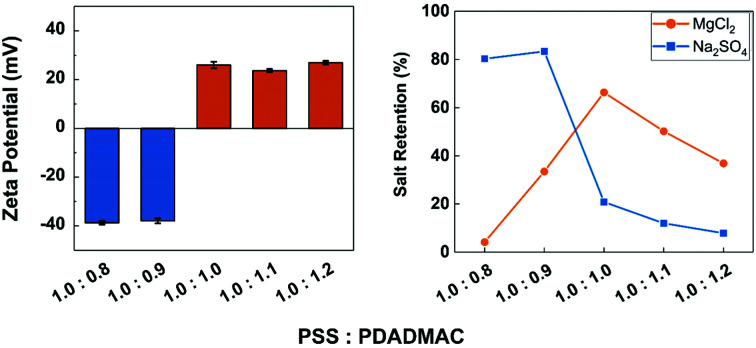

## 1. Introduction

The majority of polymeric membranes are produced using toxic and unsustainable organic solvents such as *N*-methyl-2-pyrrolidone (NMP) and dimethylformamide (DMF).^1,2^ Recently, increasing attention has been given to making membrane production processes more sustainable. The use of alternative solvents has been discussed in great detail,^3–5^ where toxic organic solvents are replaced with greener alternatives with much of this research focused on the effect of these alternative solvents on membrane structure. More recently, the Aqueous Phase Separation (APS) approach has been introduced in which polymeric membranes are formed in aqueous media, with the aim of completely eliminating the use of the organic solvents.^6,7^ Although there are earlier examples with temperature-responsive polymers,^8,9^ APS membranes are prepared from polyelectrolytes (PEs). Here, either pH-responsive PEs,^10,11^ or polyelectrolyte complexes (PECs)^7,12–14^ have been utilized. In the former, PEs are dissolved at a solution pH where they are charged, while after casting the polymer solution is immersed in a bath with a pH where the PEs are uncharged and insoluble in water.^10,11^ This process is very similar to nonsolvent induced phase separation (NIPS), where the polymer is dissolved in an organic solvent and precipitated in a nonsolvent (typically water). In APS with single PEs, water acts as both the solvent and the nonsolvent, where the only difference between the solvent and the nonsolvent is the pH of the medium (acidic *vs.* alkaline). For APS with PECs, a mixture of oppositely charged water-soluble PEs (a polycation and polyanion) is prepared such that they are not interacting each other to form a complex, then after casting, the PE mixture is immersed in a bath where the PEs can interact and form the water-insoluble complexes. Currently, there are two ways to obtain PEC-based APS membranes: 1) by using PEs where at least one of them is weakly charged, then the PE charge can be controlled *via* pH and a mixture can be prepared at a pH where the weak PE is uncharged, while the coagulation bath pH is arranged such that the PE becomes charged and interacts with the other PE to form the complex. In early work on APS, Baig *et al.* showed that a large pH change is needed, but in later work showed that with a different selection of PEs it is also possible to obtain membranes with a much milder pH change.^14^ In the salinity-change polyelectrolyte complexation approach of APS (also used in this study), the membrane preparation conditions are even milder as the PE charges are screened by a high ionic strength of the medium, while the membrane is obtained in low ionic strength coagulation baths.^7,12^ The formation of PEC-based membranes with APS is also very similar to NIPS, however, here rather than dissolving and precipitating a polymer, preventing and then allowing complexation are the key steps.

Membrane charge is a major parameter that affects separation performance.^15^ Typically, a high rejection of charged compounds can be achieved with charged membranes, and this is true even if the membrane pores are larger than the size(s) of the compounds.^16^ Here, charge exclusion dominates the rejection mechanism and it is dependent on the electrostatic repulsion forces impacted by the charged membrane. Due to attraction between charged groups of the membrane and water molecules, charged membranes are typically more hydrophilic than their neutral counterparts. This hydrophilic feature results in fouling-resistant membranes,^17^ which helps to lengthen membrane lifetime. Negatively charged membranes are even slightly more advantageous when compared to positively charged ones in terms of antifouling behavior since the majority of naturally occurring foulants are negatively charged.^18^

Obtaining charged membranes with APS from single PEs is fairly straightforward, with the charge of the PE becoming the charge of the resultant membrane.^10,11^ On the other hand, the resultant charge of membranes formed from the complexation of two oppositely charged PEs is more difficult to predict. Polyelectrolyte complexes which are prepared by mixing two PEs in a stoichiometric charge ratio are expected to have no net charge, however, in reality this is hardly ever the case.^19,20^ This effect (sometimes called ‘overcharging’) is also observed for polyelectrolyte multilayer membranes.^21,22^ For stoichiometric mixing ratios, the general tendency is that positively charged PECs are obtained;^19,23^ this is especially true for poly(sodium 4-styrenesulfonate) (PSS), and poly(diallyldimethylammonium chloride) (PDADMAC) complexes.^13,19,21,24^ Kamp *et al.*, made PSS–PDADMAC membranes by mixing the PEs in three different compositions. Even the membranes prepared from the mixture with excess polyanion (PSS) had a positive charge.^13^ Although less frequent, PSS-excess PECs have also been reported. Imre *et al.*^25^ prepared PSS–PDADMAC complexes in varying ratios and found PSS-excess PECs to have more Na^+^ ions as counterion, indicating a negative charge of the PECs. Similarly, Wang *et al.* studied the phase behavior of PSS–PDADMAC complexes and they observed that prepared coacervates could have an excess of PSS.^20^ Recently, Chen and coworkers studied the formation of PSS-overcompansated PECs when prepared with excess PSS powder in 0.1 M NaCl solutions and reported a change in material properties of PEC with changing PE mixing ratio.^26^

In this current study, we investigate the factors affecting the charge of PEC-based membranes from PSS and PDADMAC. Moreover, the effect of membrane charge on performance is also studied. PSS and PDADMAC are mixed at a high NaCl concentration in varying monomer ratios from 1.0 : 0.8 to 1.0 : 1.2 (PSS repeating unit: PDADMAC repeating unit). These casting solutions were cast and immersed in pure water baths. Resultant membranes were characterized with scanning electron microscopy (SEM), pure water permeance (PWP), molecular weight cut-off (MWCO), salt retention, and zeta potential measurements. Overall, the work will demonstrate that for APS the PE ratio is a relevant tuning parameter to determine the membrane charge, a parameter that is simply not available in traditional NIPS.

## 2. Experimental section

### 2.1. Materials

Poly(sodium 4-styrenesulfonate) (PSS, *M*_w_ ∼ 200 kDa, 30 wt% in water), poly(diallyldimethylammonium chloride) (PDADMAC, *M*_w_ ∼ 350 kDa, 20 wt% in water), poly(ethylene glycol) (PEG) of various molecular weights (200, 400, 600, 1000, 1500, and 2000 Da) were purchased from Merck. Magnesium sulfate (MgSO_4_), sodium sulfate (Na_2_SO_4_), magnesium chloride (MgCl_2_), potassium chloride (KCl) and 2-propanol (IPA) were purchased from Sigma Aldrich. Sodium chloride (NaCl, pharmaceutical grade, SanalP) was kindly supplied by Akzo Nobel.

### 2.2. Membrane preparation

In this study, membranes are prepared following procedures developed in our previous work.^12^ PSS and PDADMAC solutions were prepared at high salinity. The PSS solution had approximately 15 wt% polymer and 17.5 wt% NaCl, while the PDADMAC solution had 16.3 wt% polymer and 18.8 wt% NaCl. After these initial PE solutions became fully homogenous, they were mixed to obtain casting solutions at the desired mixing ratio of PSS to PDADMAC. This ratio will be referred to as the monomer ratio or monomer mixing ratio and it only indicates the content of the casting solutions, importantly this does not mean that the content of the resultant membranes will be in the same ratio. The casting solutions used in this study are listed in Table 1.

**Table 1:** Content of the casting solutions used for membrane preparation

PSS : PDADMAC mixing ratio	Total polymer concentration (wt%)	Total NaCl concentration (wt%)	Concentration of PSS (wt%)	Concentration of PDADMAC (wt%)	Viscosity at zero s^−1^ shear rate (Pa s)
1.0 : 0.8	15.5	18.0	9.5	6.0	5.9
1.0 : 0.9	15.5	18.0	9.1	6.4	
1.0 : 1.0	15.5	18.0	8.7	6.8	5.5
1.0 : 1.1	15.6	18.1	8.4	7.2	
1.0 : 1.2	15.6	18.1	8.0	7.6	5.2

After mixing for at least 16 hours, the casting solutions were left without stirring (for at least 20 hours) in order to remove air bubbles as these commonly cause defects in membranes. Air bubble-free, amber-colored, and transparent casting solutions with viscosities low enough to be easily processed (Table 1) were obtained. These solutions were cast on a plastic sheet with 0.3 mm casting thickness and immediately immersed in a bath containing MilliQ water (resistivity at 25 °C is 18.2 MΩ cm). After coagulation, membranes were rinsed by transferring them to washing baths which were refreshed four times in total. After the rinsing step, membranes were immersed in 30, 60, and 90 v/v% IPA baths, in order to remove the membranes from the plastic sheet without causing damage to the membrane.

### 2.3. Characterization

#### 2.3.1. Scanning electron microscopy (SEM)

Membranes were taken from the IPA and air dried overnight. Membranes maintained their opaque appearance and this is a good indication of intact porous structure (*i.e.*, the pores did not collapse during drying). Dry samples were immersed in liquid nitrogen and then broken in order to have a clear cut for cross-section images. The samples were sputter coated with a 5 nm Pt/Pd layer (Quorum Q150T ES) and were investigated by scanning electron microscopy (JSM6010LA). Cross-section images were also used to measure skin layer thickness of these membranes. Images at ×5000 magnification were analyzed with ImageJ software.

#### 2.3.2. Filtration experiments

Membrane samples were taken from IPA and washed thoroughly with demineralized water. Samples were cut to size with a 25 mm diameter hole puncher and placed in a custom-made dead-end filtration cell. The membrane effective area was 3.0 cm^2^. The pure water permeance (PWP) measurements were performed first, followed by the retention tests. For PWP tests, MilliQ water was pressurized towards the membrane and the permeate was collected as a function of time. PWP tests were continued until stable fluxes were obtained. Permeance is given by the slope of the flux against the transmembrane pressure (TMP) curve. All membranes had stable water flux at 4 bar of TMP, except for 1.0 : 1.2 mixing ratio membrane. An example for flux *vs.* TMP plot for 1.0 : 1.2 membrane is given in Fig. S1 in the ESI.† All filtration tests of the membranes were operated at 4 bar except 1.0 : 1.2 membrane which was operated at 0.5 bar. After obtaining stable fluxes, retention tests were started for the same piece of membrane sample at the same TMP.

Retention tests were conducted with either a mixture of small chain PEG molecules (for MWCO measurements) or with salts. The PEG mixture was prepared with a range of molecular weights (MWs) 200, 400, 600, 1000, 1500, and 2000 Da (1 g L^−1^ for each MW of PEG). This solution was fed to the same dead-end cell which was stirred vigorously. The initial 1 mL of permeate was discarded, and the second 1 mL of permeate sample was collected for the measurement. Feed, permeate and retentate samples were analyzed with gel permeation chromatography with a size exclusion column (Agilent 1200/1260 Infinity GPC/SEC series, Polymer Standards Service column compartment and data center (UDC 810 Interface), Polymer Standards Service Suprema 8 × 300 mm^2^ columns in series: 1000 Å, 10 μm followed by 30 Å, 10 μm)). The flow rate was 1 mL min^−1^, and the eluent was 50 mg L^−1^ NaN_3_ in MilliQ water. Retention *vs.* molecular weight (*i.e.*, the sieving curve) was plotted and the molecular weight where 90% retention was achieved is the molecular weight cut-off (MWCO) value of the membrane.^27^ There were some cases where the sieving curve reached a plateau without reaching 100% retention (Fig. S2, ESI†). When this occurs it is considered as a leak and the MWCO is approximated as the MW where the sieving curve reached 10% below the plateau. In this case, the extent of the leak was determined by the difference between 100% and the retention value of the plateau. It is important to distinguish between a leak and selectivity of a membrane. A leak is caused by defects like pinholes in the membrane and mixture that is to be separated is leaching from these defects. That is the reason the sieving curves are not reaching 100% retention even for very large molecules. On the other hand, a completely defect-free membrane would allow the permeation of some molecules and block the others. When a PEG mixture is filtered through a defect-free membrane and if size exclusion rejection mechanism is dominating, then the sieving curve is expected to reach the 100% retention. For MWCO measurements in this study, if any of them has more than 50% leak, then this was not included in the average and standard deviation calculations. For further details of MWCO measurements please refer to ref. 12.

Retention tests with salts were conducted with 5 mM MgSO_4_, MgCl_2_, Na_2_SO_4_ and NaCl solutions, separately. These solutions were fed to the dead-end cells and the measurement was performed while stirring vigorously. The solution in the cell before filtration is the feed solution, the permeate sample was collected during the filtration and the solution left in the cell is the retentate solution. Retention is calculated *via*1
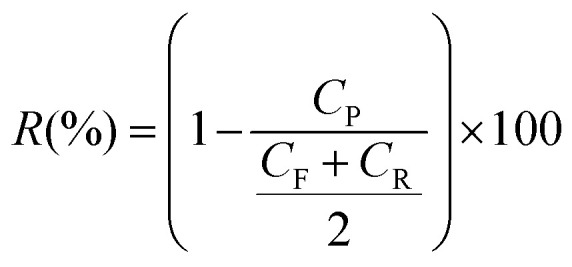
where *C*_P_, *C*_F_ and *C*_R_ are the concentrations of permeate, feed and retentate samples, respectively. Concentration of the samples were estimated by analyzing the conductivity of the solutions.

#### 2.3.3. Streaming potential

The streaming potential of the membranes was measured with an electrokinetic analyzer (Surpass, Anton Paar, Graz Austria) with an adjustable-gap cell. Prior to measurements, membranes were kept in 5 mM KCl solutions to equilibrate and 1 cm × 2 cm samples were cut. Two samples were placed in the cell such that skin layers of the samples are facing each other with a narrow slit in between. 5 mM KCl solution was pumped through the slit and the maximum pressure during the measurement is 200 mbar. A streaming current is created due to the flow of KCl solution and the zeta potential (*ζ*) is estimated by2
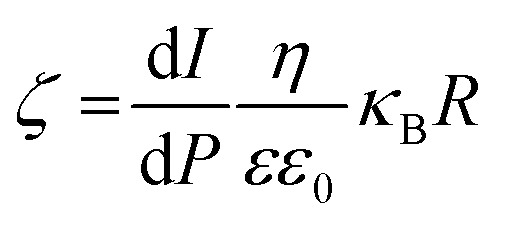
where *I* is the streaming current, *P* is pressure, *η* is dynamic viscosity, *ε* is the dielectric constant of the electrolyte solution, *ε*_0_ is the vacuum permittivity, *κ*_B_ is the specific electrical conductivity of the electrolyte solution, and *R* is the ohmic resistance of the cell. Each sample was measured three times and at least three samples were measured for all membranes. Average and standard errors are given in the following section.

## 3. Results and discussion

In our previous work, we prepared PSS–PDADMAC membranes *via* a complexation induced APS approach and the structure and performance of the membranes could be readily tuned with coagulation bath salinity, polymer molecular weight, polymer concentration, and the operation conditions.^12^ In this current article, membranes are prepared with the same method, however this time the PSS to PDADMAC monomer mixing ratio in the casting solutions has been varied from 1.0 : 0.8 to 1.0 : 1.2. We study whether the PE mixing ratio is a relevant tuning parameter to control membrane charge and thereby membrane separation properties. First, there will be a short discussion on the general membrane properties, then a more detailed discussion on the effect of monomer mixing ratio on membrane charge will take place.

Membranes from PSS–PDADMAC complexes were prepared *via* salinity change induced APS. Homogenous casting solutions were prepared at high salinity where PE interactions are screened. Five solutions at 1.0 : 0.8, 1.0 : 0.9, 1.0 : 1.0, 1.0 : 1.1, and 1.0 : 1.2 PSS to PDADMAC monomer mixing ratio were prepared. These casting solutions were immersed in MilliQ water immediately after casting. Solutions at 1.0 : 0.7 and 1.0 : 1.5 ratios were also prepared, however, these resulted in weak, gel-like films rather than solid, intact precipitates (see Fig. S3 in ESI†). Here, high charge excesses likely lead to excessive swelling of the membrane material resulting in poor material properties.^26^

**Fig. 1:**
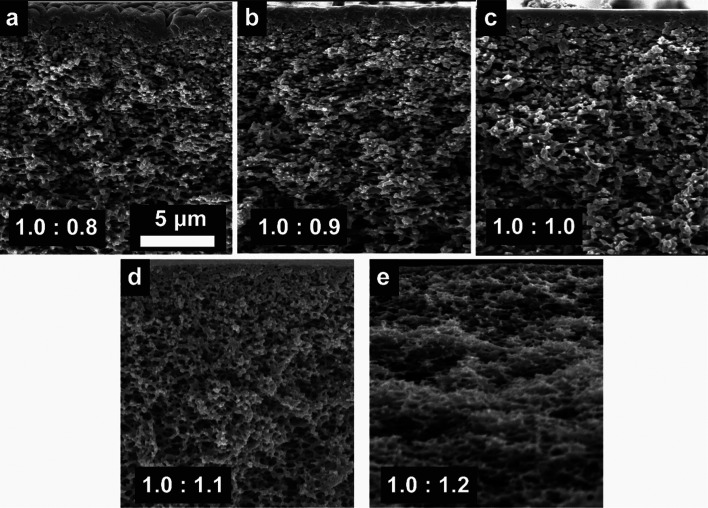
SEM images of cross-sections of membranes prepared in (a) 1.0 : 0.8, (b) 1.0 : 0.9, (c) 1.0 : 1.0, (d) 1.0 : 1.1 and (e) 1.0 : 1.2 ratios at ×5000 magnification. Images are focused on the skin layer side of the cross-section and the skin layers of the 1.0 : 1.1 and 1.0 : 1.2 membranes are thinner than 0.3 μm.

SEM cross-section images of the resultant membranes are presented in Fig. 1. Images are at ×5000 magnification and show the parts of the membranes near the skin layers of the cross-sections. It is clear that all five membranes have thin skin layers and spongy support structures without any macrovoids as seen before,^12^ which is also a very typical structure of a NIPS membrane. SEM images of the cross-sections of all the membranes studied here are given in Figure S4, and will be discussed later in this section together with filtration test results.

**Fig. 2:**
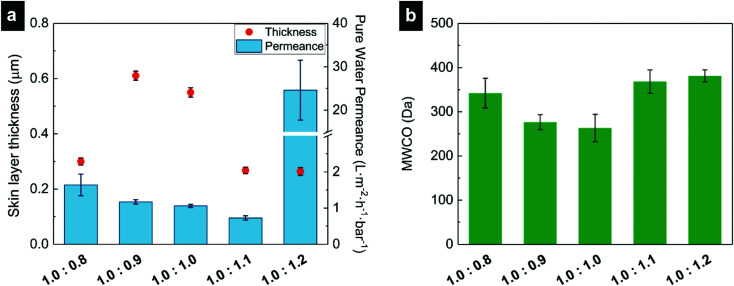
(a) Skin layer thicknesses (orange circles) and pure water permeance values (blue bars) of the membranes. Skin layer thicknesses were measured from at least eighty different points in total of three different SEM images of the same type of membrane and error bars represent the standard errors. PWP values measured at least for ten different samples and error bars represent the standard errors. Note that for the PWP axis there is a break. (b) Molecular weight cut-off values of three distinct measurements, error bars represent the standard deviations.

Fig. 2a shows the skin layer thicknesses, as determined from the SEM images, and the pure water permeance (PWP) values of the membranes. Thicker skin layers are obtained when the mixing ratio is close to 1.0 : 1.0 stoichiometry. This is relevant to observations routinely seen in NIPS processes. When sum of the polymer, solvent, nonsolvent interactions allows for nonsolvent to penetrate into the polymer solution film easier, more porous and symmetrical membranes are obtained.^1^ On the other hand, when polymer precipitates immediately at the membrane surface, the emerging skin layer hinders the diffusion of the solvent and the nonsolvent, which leads to asymmetrical membranes with distinct skin layers.^1^ In our work, the membranes prepared using the close to stoichiometric ratio PE mixtures have lower water contents than the membranes made from nonstoichiometric mixtures. The lower water content results to stiffer PECs^26^ and developing a skin layer hinders permeation of the polymer, water and salt. Moreover, Fig. 2a shows a decrease in PWP as the concentration of the PDADMAC in the casting solution increases; except for the 1.0 : 1.2 membrane where a very high PWP is found. To check reproducibility, we prepared membranes following the same procedure at least four times and the structure of these membranes were imaged with SEM each time. All of the cross-section SEM images are given in Fig. S4 (ESI†). As can be seen in the figure, the membrane structures were mostly reproducible, but occasionally thicker skin layers were found. These thicker layers are likely the result of pore collapse during the drying process required for SEM imaging and are considered as outliers and were not included in Fig. 2a. Supporting this hypothesis is the fact that the PWP values have relatively small error bars (*i.e.*, a high reproducibility, in line with our previous study regarding the 1.0 : 1.0 membranes^12^); these membranes were never dried unlike the samples for SEM measurements. The error bars of the PWP values of the 1.0 : 0.8 and 1.0 : 1.2 membranes are somewhat larger than the others and these membranes are also softer and more swollen than the others (Fig. S5, ESI†). Charges attract water molecules and if one of the PE is in excess, there will be a higher affinity for water molecules which leads to plasticizing effect for PECs.^24,26,28–30^ This results in softer and more swollen membranes and less reproducible measurements.

The molecular weight cut-off (MWCO) of the membranes was also measured. Fig. 2b shows the MWCO values of the resultant membranes and it is lower than 400 Da for all cases, even for the 1.0 : 1.2 membranes that have a high PWP. This indicates that these films are indeed membranes (*i.e.* permeate selectively) and that they all perform as nanofiltration membranes.^1^ MWCO values of the 1.0 : 0.9 and 1.0 : 1.0 membranes are lower than the other membranes, which indicates that they are tighter (smaller average pore size) than the others. This can again be related to the effect of mixing ratio on material properties of the membranes. With a lower excess of charge, these membranes would be expected to be less swollen in water. Achari *et al.* and Chen *et al.* reported that the PECs that are closer to the stoichiometric ratio have a higher *T*_g_ due to a lower degree of water plasticization.^26,31^ The increasing number of ionic cross-links makes the PE chains less mobile and the PEC less permeable (*i.e.* denser). This is also consistent with the observations for PEMs, in which more swollen layers have higher permeances.^32^ Moreover, it is worth noting that small defects were observed with MWCO measurements, in line with our previous study (see Table S1 and Fig. S2 in ESI†). It is speculated that the polydispersity of the PEs cause these defects as the phase separation behavior of the polymer chains is different for different chain lengths.^12,33^ Because the polydispersity of the PEs was the same as our previous study, these defects were expected. However, less leakage was observed for 1.0 : 0.8 and 1.0 : 1.2 membranes when compared to other mixing ratio membranes. This is possibly due to more swollen and softer nature of these membranes. The more the membranes are doped with water, the more mobile the polymer chains will be, leading to more resistive to defect formation. Overall, Fig. 2 clearly indicates that the obtained films are nanofiltration membranes making it interesting to study them for their retention of ions.

**Fig. 3:**
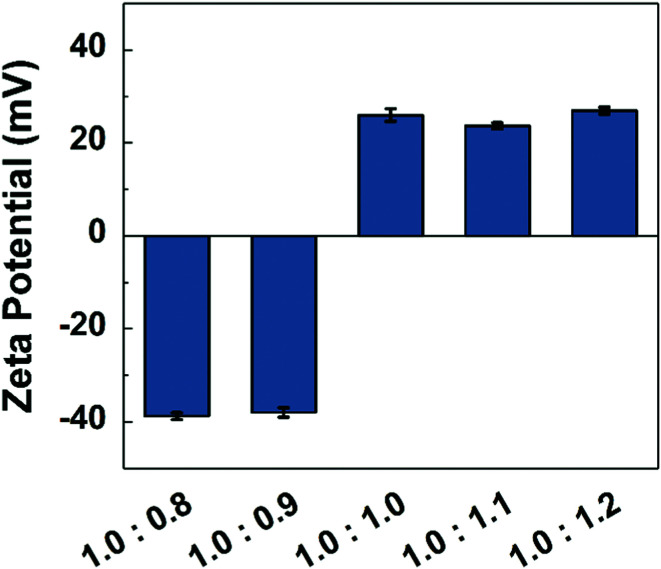
Zeta potential of the membranes prepared in various mixing ratios in the casting solutions. At least three samples of each membrane were tested, each sample was measured minimum three times and a measurement gives four data points. Blue bars represent the average of these data points and error bars represent the standard errors. pH was measured as 5.8 ± 0.1 during the tests.

The next step was to investigate whether PE mixing ratio is an effective parameter to control membrane charge. To study this, we measured the streaming potential of these membranes. Here, a 5 mM KCl solution flows through a narrow channel where the walls are two membranes with the skin layers facing the channel. The electric potential that results from this flow was measured as streaming potential which is directly related to the zeta potential of the membranes (see eqn (2)). The results are given in Fig. 3 and clearly shows the charged nature of all the membranes. Even the 1.0 : 1.0 solution led to positively charged membranes, possibly indicating some leakage of PSS into the coagulation bath. Although counter-intuitive, overcharging of PECs is observed^34^ and positively charged PSS–PDADMAC complexes^13,19,24^ and multilayers^21,22^ are frequently reported in literature. Membranes from 1.0 : 1.0 solutions and the solutions that have excess PDADMAC give positively charged membranes, while membranes from 1.0 : 0.9 and more excess PSS are negatively charged. It is expected that the solutions that have a larger excess of one of the PEs will have higher charge densities. However, this is not observed, where the zeta potentials are rather stable except for the flip between 1.0 : 0.9 and 1.0 : 1.0. This likely stems from the swelling of the membranes that also increases with a higher charge density.^26^ More swelling dilutes the density of the charges and leads to a lower measured zeta potential. Our observations during membrane formation and during the handling for filtration tests confirms this expectation. Both 1.0 : 0.8 and 1.0 : 1.2 membranes are significantly softer than the other membranes and they are more swollen during the coagulation process (see Fig. S3, ESI†). Our results clearly show that it is possible to change the charge of the membrane with PE mixing ratio. There is a clear point in the mixing ratio (between 1.0 : 0.9 and 1.0 : 1.0) where the resultant membrane charge flips between positive and negative which is a unique observation to this study. Krishna *et al.*, prepared dense membranes by hot-pressing PSS–PDADMAC complexes and varied the molecular weight (MW) of the PEs. All combinations resulted in positively charged membranes.^35^ Kamp *et al.* prepared PSS–PDADMAC membranes with complexation induced APS, where they used solutions in three different ratios (1.0 : 0.8, 1.0 : 1.0, 1.0 : 1.2). All the resultant membranes had an effective positive charge.^13^ At first glance, the approach of Kamp *et al.* and this work looks fairly similar and there is no apparent reason to expect different membrane charges and structures. However, when we look in detail, there are clear differences in PE molecular weight (PSS in this work 200 kDa, in the work of Kamp *et al.* 1000 kDa; PDADMAC: 350 kDa *vs.* 500 kDa), total polymer concentration (15.5 wt% *vs.* 25 wt%) and salt type (NaCl *vs.* KBr).^13^ PE molecular weight cannot be the reason for the difference in results since Krishna *et al.* obtained positively charged PECs in all MW combinations.^35^ Differences in polymer concentration could be a reason for differences in membrane structures, but it is a less likely reason for the differences in membrane charge. However, salt type is a very important parameter for the structure of PECs. In NIPS, thermodynamics and kinetics are the two major factors that determine membrane structure and performance^1,27,33^ and it is already known that there are strong similarities between APS and NIPS processes.^13,36^ In literature, there are many studies that have reported that KBr is a better doping agent than NaCl for PSS–PDADMAC complexes,^20,34,37–39^ this means that the solvent quality of KBr is higher than NaCl for PSS–PDADMAC complexes. Moreover, it is known that different salts have different diffusion rates within PECs,^37^ which would also affect the phase separation kinetics. Therefore, a simple change in salt type results in both a change in thermodynamics and kinetics, which could allow a change in membrane structure, performance, and charge. Unlike KBr, NaCl is the salt for PSS–PDADMAC complexes that prevents expulsion of PSS to coagulation bath, which leads to formation of negatively charged membranes. The correct choice of salt type is thus essential in APS to make good use of the PE ratio as a tuning parameter to control the membrane charge. One of a few instance that we came across in literature for PSS-excess PECs was a study of Imre *et al.*^25^ and Oyama *et al.*^40^ also Na^+^ and Cl^−^ were used as counterions. Recently, Chen *et al.* also obtained these kinds of PECs which are also immersed in dilute NaCl solutions.^26^

**Fig. 4:**
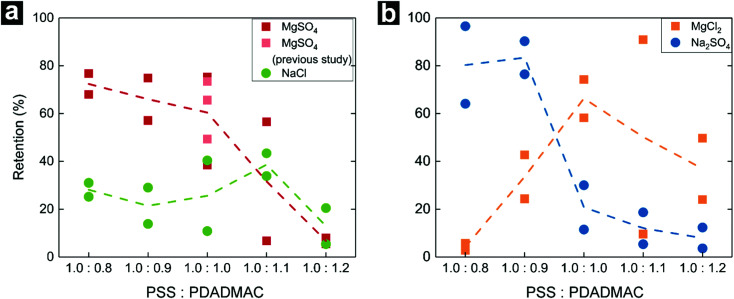
Salt retentions of the membranes. Each symbol represents a measurement. Dashed lines are the average of the measurements given to guide the eye. (a) Red and pink squares are MgSO_4_ retentions of this study and previous study,^12^ respectively. (b) green circles are for NaCl retentions, orange squares are for MgCl_2_ retention and blue circles are for Na_2_SO_4_ retentions.

Lastly, the effect of membrane charge on the separation of charged components was investigated. Individual samples of the membranes were subjected to retention tests of 5 mM MgSO_4_, NaCl, MgCl_2_, and Na_2_SO_4_ solutions. Retention values of the membranes for symmetric salts (divalent–divalent or monovalent–monovalent) are given in Fig. 4a, and for asymmetric salts (divalent–monovalent or monovalent–divalent) in Fig. 4b. Retention values of symmetric salts are very similar to each other, except for the highly permeable 1.0 : 1.2 membranes and MgSO_4_ retentions are always higher than NaCl retentions. On the other hand, for asymmetric salts retention is varying depending on the membrane mixing ratio. While 1.0 : 0.8 and 1.0 : 0.9 membranes are able to retain the salt containing a divalent anion (Na_2_SO_4_) better, 1.0 : 1.0, 1.0 : 1.1 and, 1.0 : 1.2 membranes retain the salt that has a divalent cation (MgCl_2_) better. This is very typical for separation mechanisms dominated by Donnan exclusion which applies for charged membranes.^1,2,21,41^ Simply, the ions that have a higher valence are repelled more by the similarly charged membranes, therefore these ions are retained more during filtration. The retention results follow exactly the same trend as the streaming potential measurements confirming that 1.0 : 0.8 and 1.0 : 0.9 membranes are negatively charged while 1.0 : 1.0, 1.0 : 1.1, and 1.0 : 1.2 membranes are positively charged.

For all measurements, retention performance of 1.0 : 1.2 membrane is the lowest. As PWP tests indicate, these membranes are the most permeable of all due being highly swollen by water, therefore, this membrane might have a more loose structure when compared to others. The relatively low ion retentions, coupled to the low MWCO (Fig. 2) make this membrane quite relevant for separations where one would like to stop organic molecules while allowing salts to permeate.^42^

Considering that the 1.0 : 0.9 and 1.0 : 1.0 membranes have similar performance to the 1.0 : 0.8 and 1.0 : 1.1 membranes, respectively, and that these membrane are actually more mechanically stable (1.0 : 1.0 membranes are even stable at 10 bar pressure for more than 50 hours^12^), it is logical to choose the 1.0 : 0.9 or 1.0 : 1.0 membranes over the other ones studied here. Therefore, we can conclude that a little excess PE in the casting solution is more than enough to change the membrane charge and performance, while a larger excess reduces the mechanical properties of the membranes. Still, the 1.0 : 1.2 membrane is very interesting because of the high water permeance and the interesting combination of a low MWCO and low ion retentions. Combining membranes with good mechanical properties, with a thin layer of membranes with these interesting separation properties could be possible through double casting knives^43^ or triple spinnerets.^44^

## 4. Conclusion

Five different casting solutions were prepared by mixing PSS and PDADMAC solutions at high salinity. The monomeric ratio of PSS to PDADMAC was varied from 1.0 : 0.8 through to 1.0 : 1.2. The mixed solutions were cast and immersed in MilliQ water baths leading to membrane formation. The structure, charge, and filtration performance of the membranes was studied. All membranes had similar morphologies with thin skin layers and porous support layers without the presence of macrovoids. When PSS was in excess in the casting solutions, the membranes were negatively charged, and when the solutions were in a stoichiometric ratio or with excess PDADMAC, the membranes were positively charged. This possibility to tune membrane charge by controlling PE ratio is reported for the first time and we argue that this is because of the role salt type plays. Unlike other papers in the literature, here we use NaCl which is a different dopant for PSS–PDADMAC complexes than KBr. The different salt type leads to differences in the thermodynamics and kinetics of the phase separation and results in differences in membrane structure and charge. It is likely that NaCl allows trapping of the excess polymer chains, while in KBr system the additional mobility would allow chain expulsion of PSS.

While the negatively charged membranes retained Na_2_SO_4_ better, the positively charged membranes had a higher retention for MgCl_2_, indicating a separation mechanism dominated by Donnan exclusion. The PE mixing ratio also affects the material properties of the membranes. Here, as the PE excess increased, the resultant membranes became more plasticized and swollen by water. Consequently, these membranes were more permeable, yet they were less stable against applied transmembrane water pressure. The 1.0 : 0.9 and 1.0 : 1.0 membranes performed well and had the lowest MWCO value, high salt retentions, and good mechanical stability. Therefore, there is no need to have casting solutions with large PE excesses unless membrane swelling and softness is desired. It takes only a small excess of a PE to completely change membrane separation performance. Still, for the larger excess of 1.0 : 1.2 a very high water flux was obtained, with an interesting combination of low MWCO and low ion retentions. This opens up new possibilities in the preparation of PEC-based membranes where stoichiometric ratios are used for the mechanical properties, combined with an excess charge system that provides interesting separation properties.

## Author contributions

Elif Nur Durmaz: conceptualization, data curation, investigation, methodology, writing-original draft. Joshua D. Willott: data curation, investigation, writing-review and editing, supervision. Md Mizanul Haque Mizan: data curation, investigation, methodology, writing-review and editing. Wiebe M. de Vos: conceptualization, project administration, supervision, writing-review and editing, funding acquisition.

## Conflicts of interest

There are no conflicts to declare.

## Supplementary Material

SM-017-D1SM01199E-s001
