# Supplementary material for: Tuning the charge of polyelectrolyte complex membranes prepared via aqueous phase separation
Source: Soft Matter. 2021 Sep 30;17(41):9420–7. doi: 10.1039/d1sm01199e (PMC8549507; doi:10.1039/d1sm01199e)
Supplement: SM-017-D1SM01199E-s001 [file SM-017-D1SM01199E-s001.pdf]

Electronic Supplementary Material (ESI) for Soft Matter.
This journal is © The Royal Society of Chemistry 2021

Supplementary Information

Tuning the Charge of the Polyelectrolyte Complexes Membranes Prepared via Aqueous Phase Separation

Elif Nur Durmaz, Joshua D. Willott, Md Mizanul Haque Mizan, Wiebe M. de Vos*

Membrane Science and Technology, MESA+ Institute for Nanotechnology, University of Twente, Faculty of Science and Technology, P.O. Box 217, 7500 AE Enschede, the Netherlands

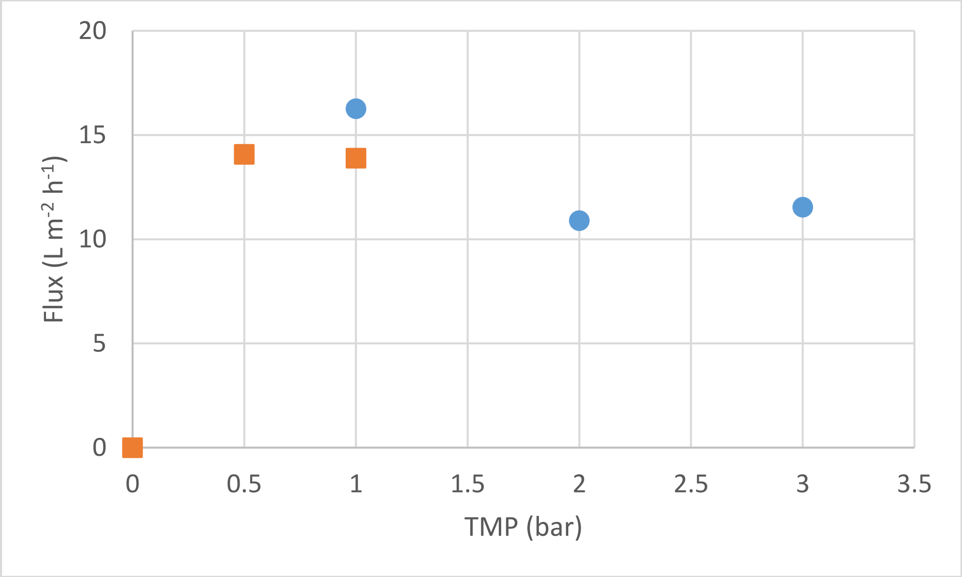

Figure S1. Flux against transmembrane pressure plot of 1.0:1.2 membrane. Results for two distinct samples showing that this membrane is not stable pressures higher than 0.5 bar.

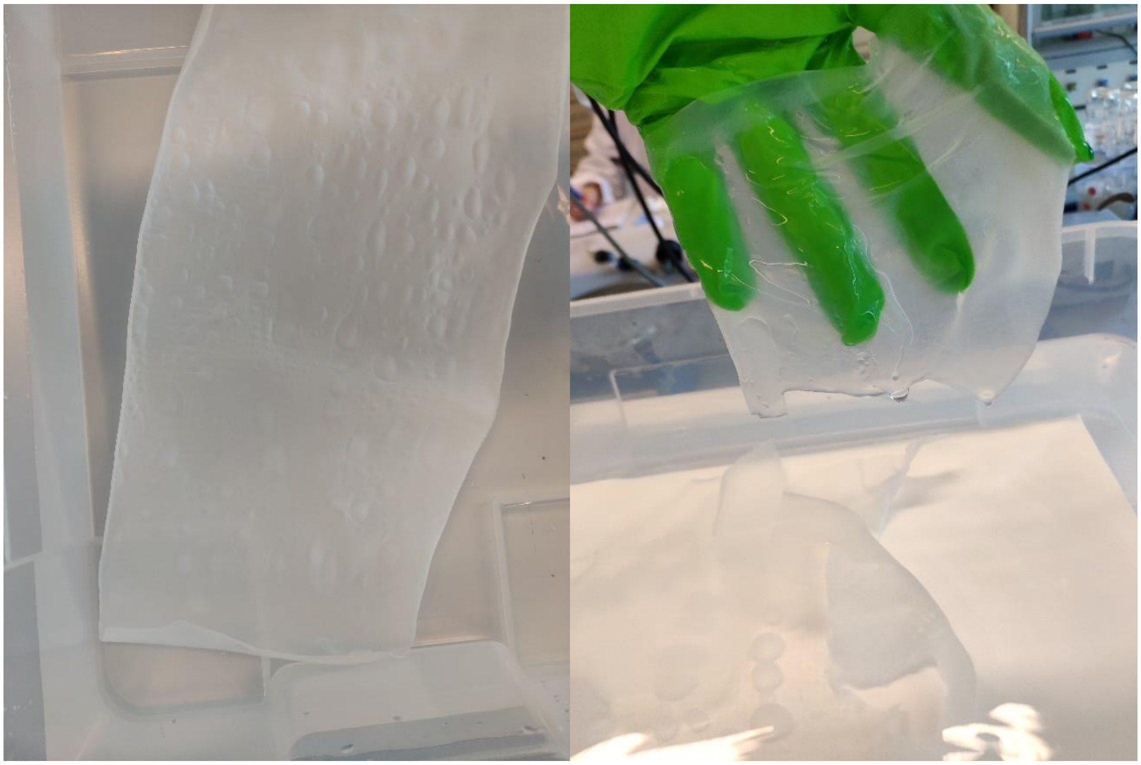

Figure S2. PSS-PDADMAC solution in 1.0:0.7 (left) and 1.0:1.5 (right) ratio was cast an immersed in MilliQ water. 1.0 0.7 films was cast in the size of approximately 2/3 of the one seen in the photograph, during coagulation it got swollen. 1.0:1.5 film was translucent and gel-like due to being highly swollen and this led to very weak films.

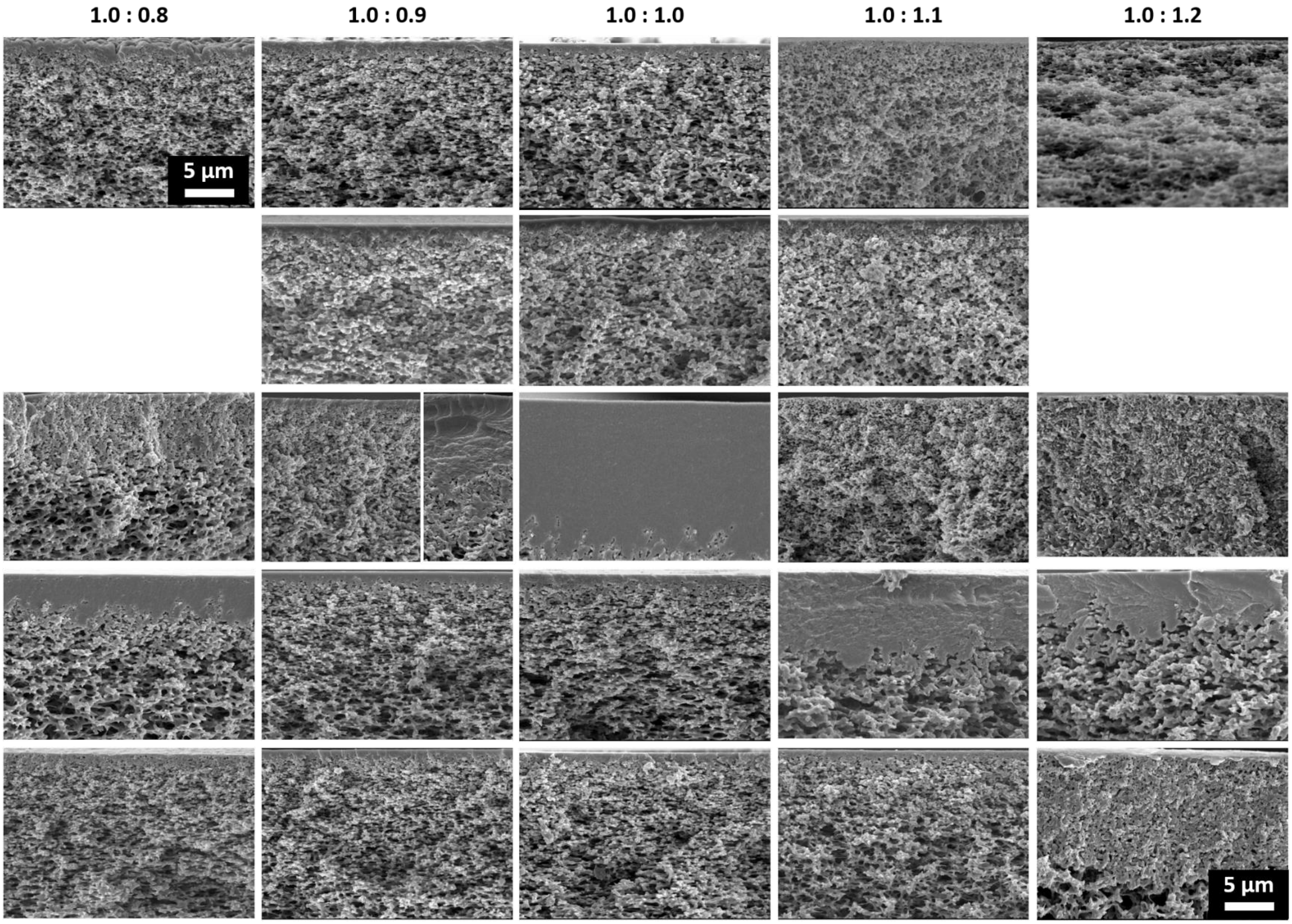

Figure S3. SEM images of cross–sections of membranes prepared in varying ratio at ×5000 magnification. Images are focused on the skin layer side of the cross–section. Each row represents one set of solutions prepared and precipitated at the same time. All membrane sets were prepared in exactly same way. There are two images in the third row of the 1.0:0.9 membranes. Both images belong to the same membrane sheet, however samples were dried at different times. Skin layers larger than 5 µm are not included in Figure 1c.

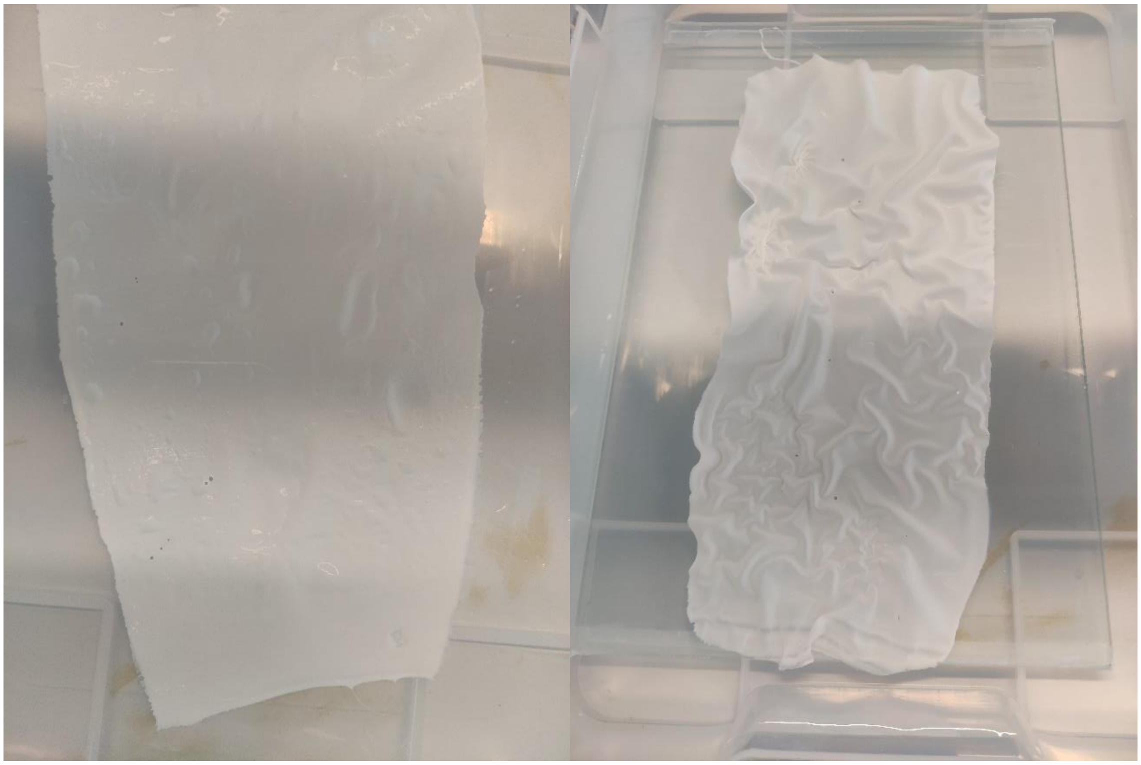

Figure S4. PSS-PDADMAC 1.0 0.8 (left) and 1.0 1.2 (right) membranes. Bumps and wrinkles are due to swelling. Both membranes were cast in smaller sizes and they got swollen during coagulation. When compared to each other, 1.0 1.2 membranes are more swollen and softer.

Table S1. Molecular weight cut-off and extent of leaks during filtrations of PEG solutions.

**Mixing Ratio**	**Molecular Weight Cut-off**	
	Cut-off (Da)	Leak (%)
1.0 0.8	340 ± 30	8 ± 3
1.0 0.9	280 ± 20	20 ± 10
1.0 1.0	260 ± 30	20 ± 15
1.0 1.1	370 ± 30	20 ± 10
1.0 1.2	380 ± 10	15 ± 10
